# Methotrexate-induced acute cardiotoxicity requiring veno-arterial extracorporeal membrane oxygenation support: a case report

**DOI:** 10.1186/s13256-022-03644-9

**Published:** 2022-11-29

**Authors:** Sareena Shah, Kristen Haeger-Overstreet, Brigid Flynn

**Affiliations:** 1 University of Missouri-Kansas City School of Medicine, 2411 Holmes, Kansas City, MO 64108 USA; 2 Department of Pharmacy, The University of Kansas Health System, 4000 Cambridge St., Mail Stop 4040, Kansas City, KS 66160 USA; 3 Department of Anesthesiology, University of Kansas Health System, Mail Stop 1034, 3901 Rainbow Blvd., Kansas City, KS 66160 USA

**Keywords:** Methotrexate toxicity, Acute cardiotoxicity, Leucovorin rescue

## Abstract

**Background:**

Methotrexate is an antifolate antimetabolite that inhibits the activity of dihydrofolate reductase by acting as a false substrate, which leads to defects of DNA synthesis, specifically the inhibition of purine and pyrimidine synthesis. Thus, methotrexate is a powerful agent for treating autoimmune diseases and cancer. In general, methotrexate is thought to be cardioprotective and reports of methotrexate-induced cardiomyopathy are rare. We present a case of methotrexate-induced severe cardiotoxicity diagnosed by exclusion of all other potential causes.

**Case presentation:**

The patient was a 54-year-old Caucasian man presenting to an outside hospital with a chief complaint of abdominal pain and bloating who reported taking methotrexate up to 20 mg per week for systemic sclerosis. After a transthoracic echocardiogram found a left ventricular ejection fraction of 10% and coronary catheterization demonstrated no significant disease, he was transferred to our hospital for advanced heart failure therapies. His condition deteriorated, and he was eventually placed on veno-arterial extracorporeal membrane oxygenation. Owing to a lack of an identifiable etiology of cardiac failure, toxicology consultation recommended 24 hours of intravenous leucovorin therapy to overcome any residual and potentially cardiotoxic methotrexate still in his system. Over the next 5 days, his cardiac function improved daily, such that on day 5 of extracorporeal membrane oxygenation, he had a left ventricular ejection fraction of 40% and was able to be decannulated. Two days later, his ejection fraction improved to 60% and normal right ventricular function. Initially, his renal function improved while on extracorporeal membrane oxygenation, but over the next week deteriorated such that he required intermittent hemodialysis until hospital discharge.

**Conclusions:**

After a process of elimination, the most likely cause of this patient’s acute decline and rapid recovery of bi-ventricular function was methotrexate toxicity. Leucovorin may have aided the reversal of methotrexate toxicity.

## Introduction

Methotrexate (MTX) is an antifolate antimetabolite, commonly prescribed for tumoral pathologies, such as acute lymphoblastic leukemias, and in rheumatology for rheumatoid polyarthritis and other chronic inflammatory diseases [1]. Methotrexate inhibits the activity of dihydrofolate reductase by acting as a false substrate, which leads to defects of DNA synthesis, specifically purine and pyrimidine synthesis inhibition [1]. These attributes make MTX a powerful agent targeting autoimmune diseases and cancer. However, MTX is also associated with numerous toxicities, including nausea, vomiting, diarrhea, myelosuppression, pancytopenia, liver dysfunction, acute renal failure, pulmonary symptoms, mucositis, stomatitis, ulceration/erosion of the gastrointestinal system, and cutaneous ulcerations [2]. In general, MTX is thought to be cardioprotective [3], and reports of MTX-induced cardiomyopathy are very rare and typically involve high doses or dosing errors [1]. After obtaining patient consent, we present a case report of MTX-induced severe, acute cardiomyopathy requiring veno-arterial extracorporeal membrane oxygenation (VA-ECMO) with low-to-normal drug levels.

## Case description

A 54-year-old Caucasian man presented to an outside hospital with the chief complaint of abdominal pain and bloating for which he had been taking nonsteroidal antiinflammatory drugs (NSAIDs). His medical history included hypertension, Raynaud’s syndrome, rectal cancer in remission following surgical resection and treatment with radiation therapy, and ongoing radiation-induced cystitis. He was also diagnosed 2 months prior with strongly positive anti-RNA poly III systemic sclerosis following new onset dysphagia and was prescribed MTX until he reached a dose of 20 mg every week. However, he had not taken MTX in 14 days since he began feeling ill. He was found to have a small rupture of his bladder due to cystitis, which was deemed to not require surgical intervention. During the course of the bladder workup, he was found to have abnormal serum sodium and creatinine levels (Table 1), prompting and echocardiogram. A transthoracic echocardiogram revealed a severely reduced left ventricular (LV) ejection fraction of 10% that led to a coronary catheterization. This demonstrated no significant coronary disease. He was subsequently transferred to our hospital for advanced heart failure therapies.

**Table 1:** Notable laboratory values present on admission. Of note, his liver and renal function tests were normal a month prior to his admission

BNP	> 5000 pg/mL (upper limit of test)
Troponin	> 22 ng/mL (upper limit of test)
Lactic acid	9 mmol/L
Sodium	117 mmol/L
AST	7945 U/L
ALT	4996 U/L
Creatinine	3.78 mg/dL
White blood cells	17.7 × 10^9^
Platelets	547 × 10^9^

*BNP* brain natriuretic peptide, *AST* aspartate aminotransferase, *ALT *alanine aminotransferase

**Fig. 1:**
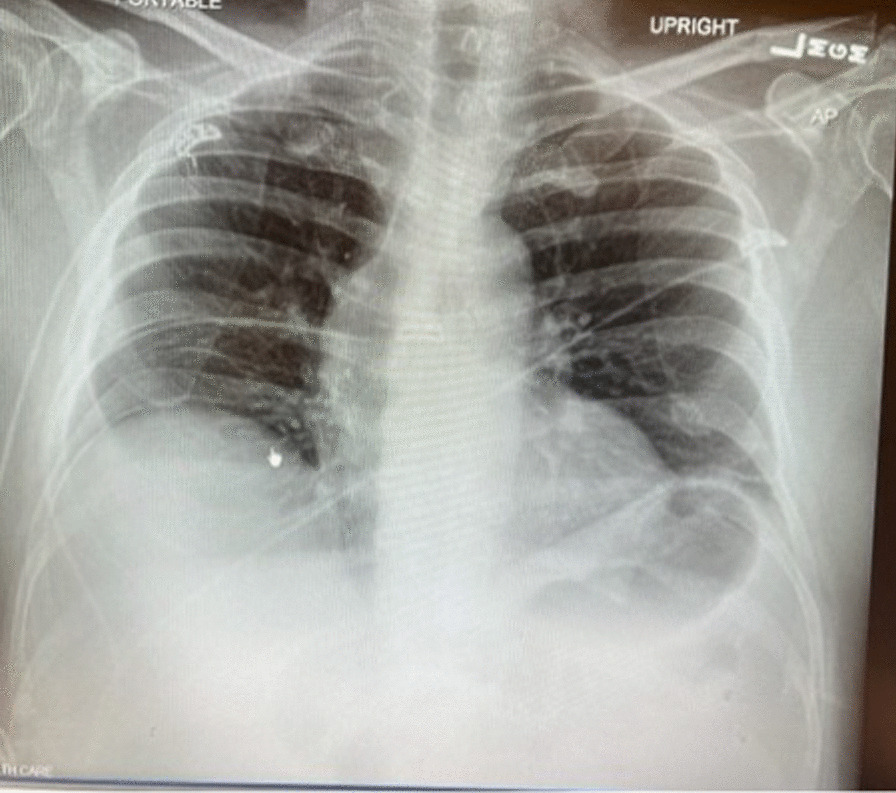
Chest X-ray upon admission showed perivascular congestion and gastric air. Cardiomegaly or significant pleural effusions were not present. Mediastinal structures appeared normal

Upon arrival, he was conversational and pleasant. His physical examination was significant for sclerodactyly and abdominal ascites. His blood pressure revealed a narrow pulse pressure of 93/82 mmHg with a heart rate of 94 bpm and he was afebrile. His admission chest X-ray was unremarkable (Fig. 1). His bedside echocardiogram demonstrated LV ejection fraction of 10%, LV size of 4.3 cm, LV wall width 1 cm, and severe right ventricular failure. Intravenous dobutamine of 5 mcg/kg/minute was initiated, which shortly thereafter required addition of intravenous norepinephrine to maintain a mean arterial blood pressure of 65 mmHg. Laboratory analysis demonstrated severe cardiac failure (Table 1). His viral respiratory panel was negative for 19 common viruses. His chest X-ray was unremarkable. A pulmonary artery catheter was placed revealing right atrial pressure of 10 mmHg and pulmonary pressures of 20/10 (14) mmHg and pulmonary artery occlusion pressure of 14 mmHg, indicating he was not in a hypervolemic or pressure overloaded state.

The patient stated that he attempted to exercise, however, this was limited by skin contractures due to systemic sclerosis. He had been in his usual state of health working daily and walking until approximately 2 weeks prior to his admission. He revealed he had been diagnosed with COVID a month prior but only had mild symptoms that seemed resolved. He did not experience shortness of breath or other heart failure symptoms until approximately 9 days prior to admission. He confirmed that he was recently initiated on oral methotrexate for worsening systemic sclerosis symptoms, especially those involving the skin. He had been vigilant concerning his dosing regimen until stopping MTX owing to not feeling well in the setting of COVID.

During the initial evaluation, his mental status waned to somnolence and lethargy. After discussion with the patient and his wife that he would not survive with this degree of cardiomyopathy and the ensuing end-organ damage, he was placed on VA-ECMO via right internal jugular and right femoral arterial cannulation. Hemodynamic infusions included dobutamine, epinephrine, norepinephrine and vasopressin. He was initiated on bolus steroids for presumed viral myocarditis.

**Fig. 2:**
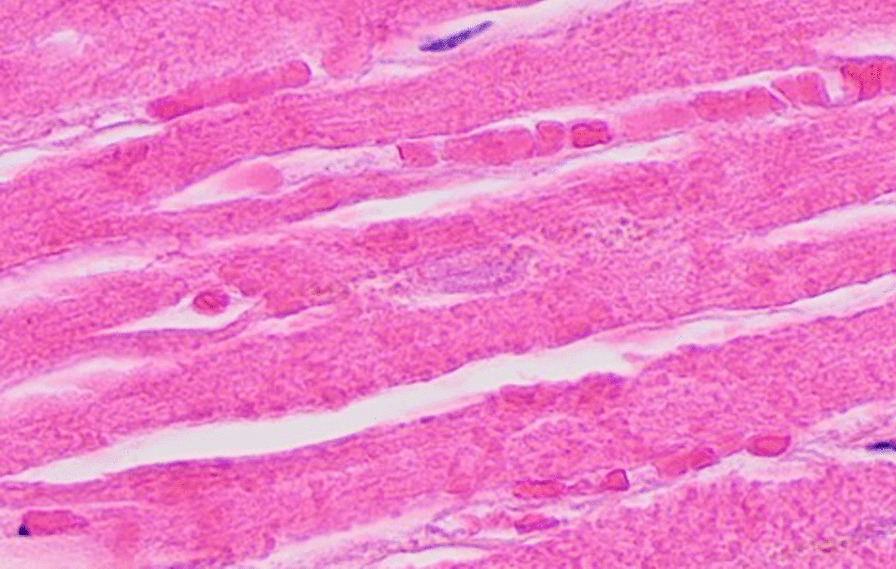
Histopathology demonstrating myocardial necrosis. Myocardial cells with H and E stain demonstrate loss of nuclei with only one barely visible nucleus remnant in the center. *FH *dihydrofolate, *dTMP *thymidylate, *dUMP* deoxyuridine monophosphate. Image by Mikael Häggström, MD, used with permission. https://commons.wikimedia.org/w/index.php?curid=109899804

**Fig. 3:**
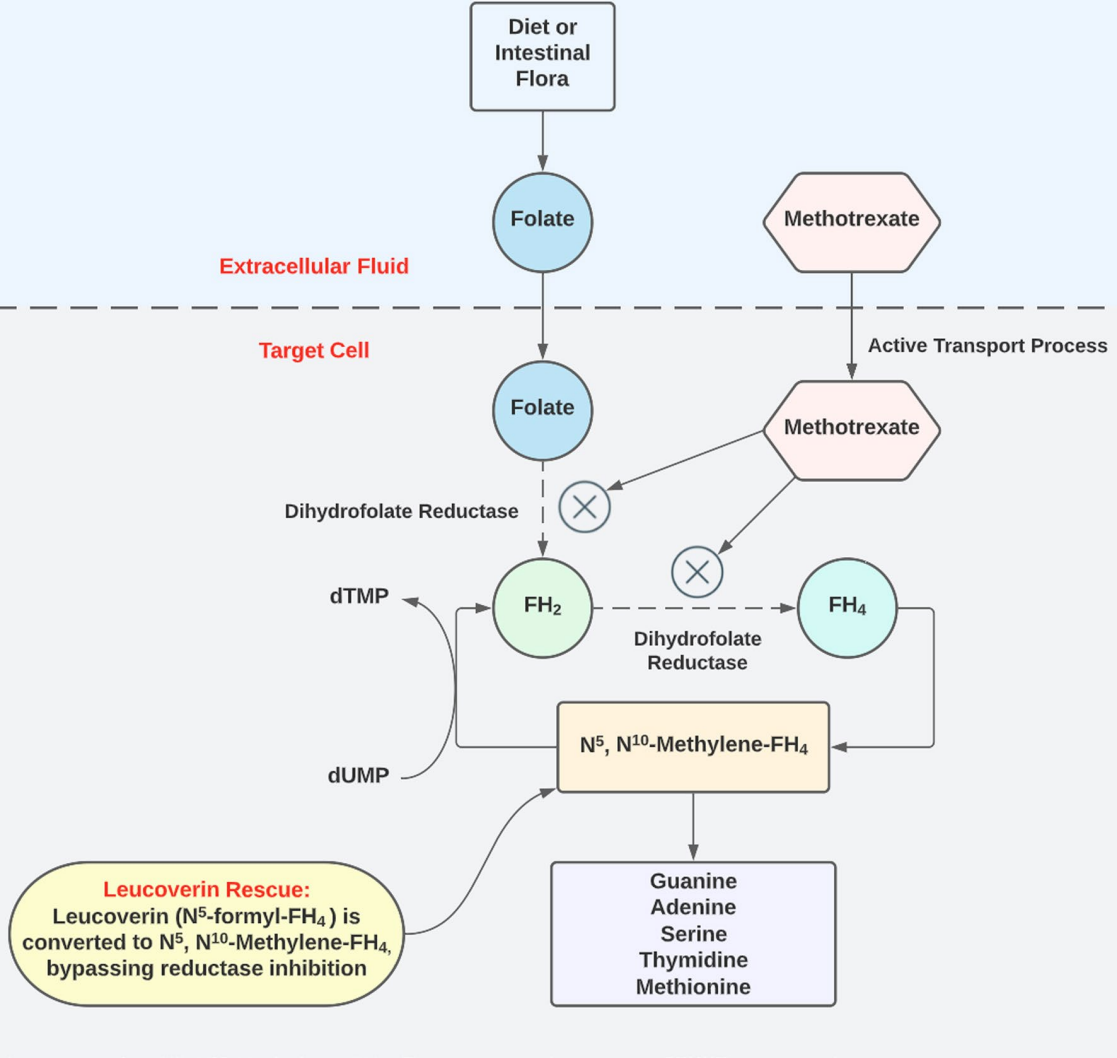
Methotrexate acts as a false substrate and inhibits dihydrofolate reductase, leading to defects in DNA synthesis, specifically the inhibition of purine and pyrimidine synthesis. This action can be bypassed by leucovorin, allowing for DNA synthesis

Etiologies considered for his severe cardiomyopathy included viral myocarditis, systemic sclerosis, COVID-induced cardiac dysfunction, and lastly, methotrexate toxicity. His MTX level was very low (< 0.04 μmol/L) being checked after discontinuing MTX for 14 days. A cardiac biopsy was obtained and showed cardiac necrosis (Fig. 2) without evidence of inflammation or fibrosis, indicating myocarditis was not present. Thus, steroids were stopped due to risk of scleroderma renal crisis. Owing to the lack of an identifiable etiology of cardiac failure, toxicology consult recommended 24 hours of intravenous leucovorin therapy to overcome any residual and potentially cardiotoxic MTX still in his system (Fig. 3).

Over the next 5 days, his cardiac function improved daily such that on day 5 of ECMO, he had an LV ejection fraction of 40% and was able to be decannulated. Two days later, his ejection fraction improved to 60% and normal right ventricular function. Initially, his renal function improved while on ECMO, but over the next week, deteriorated such that he required intermittent hemodialysis until hospital discharge. Notable medications at discharge included oral methotrexate 20 mg weekly, oral folic acid, and oral carvedilol. At 6-month follow-up, the patient was in a rehabilitation center, off hemodialysis, and gaining strength in hopes of returning home.

## Discussion

The etiology of this patient’s acute cardiac failure required a multidisciplinary approach. While several etiologies were considered, the authors were able to eliminate all except MTX-induced cardiotoxicity as the cause of this patient’s sudden cardiac decline.

Therapeutic MTX prevents the body from using folic acid by inhibiting dihydrofolate reductase, resulting in inhibition of DNA synthesis, repair, and cellular replication, leading to cell death. Organs most often affected by MTX toxicity are the bone marrow (leukoneutropenia and thrombocytopenia), lung (pneumonitis), kidney (precipitation of MTX metabolites), brain (encephalopathy), and liver (hepatitis to fulminant liver failure). Cardiotoxicity due to MTX is extremely rare, with only one previous case report attributing MTX toxicity to cardiomyopathy [1]. Additionally, oral mucosal lesions are one of the first signs of MTX toxicity, which our patient did not have. Our patient also did not have any signs of bone marrow suppression, actually having leukocytosis and mild thrombocytosis.

By the time our patient arrived at our institution, his MTX level was nearly undetectable; however, he had not taken MTX for 14 days. Therefore, it is unknown what his peak level was while taking the drug. Without other probable etiologies, given the cardiac biopsy and owing to low risk, the decision was made to initiate intravenous leucovorin 100 mg/m^2^ every 6 hours for 24 hours. Leucovorin, or folinic acid, acts as folic acid in vivo. Leucovorin bypasses the enzymes that are inhibited by MTX, thereby allowing nucleic acid synthesis to proceed even in the presence of methotrexate. Leucovorin can also compete with methotrexate for the same transport processes into the cell [4].

Contrary to findings in the current report, several clinical trials have shown that MTX is associated with improved endothelial function, slower atherosclerosis progression, decreased risk of major cardiovascular adverse events, and benefits on cardiovascular survival [3, 5]. The beneficial cardiovascular effects of MTX, although incompletely understood, are explained by its antiproliferative, immunosuppressive, antiinflammatory, and antiatherogenic effects. While it seems that MTX has favorable effects on cardiopulmonary endothelium, it also promotes cell death, possibly of cardiac myocytes. In rare patients, such as the current case presentation, patients may develop cardiotoxicity. Indeed, our patient had normal coronary, valvular, and pulmonary vasculature, juxtaposed with severe cardiomyopathy and myocardial necrosis on cardiac biopsy.

MTX metabolism varies based on the route of administration. When given orally, MTX will undergo hepatic metabolism and some intestinal metabolism. When given intravenously, up to 90% of the drug is excreted unchanged in the urine. The optimal oral dose (maximum 25 mg/week) should be reached within 4–8 weeks. The digestive absorption of MTX is variable because 45–55% of MTX is protein bound. Thus, undernutrition will increase free MTX. Other risk factors for MTX toxicity include high doses of MTX, renal impairment, nonsteroidal antiinflammatory drugs, age over 55 years, folate deficiency, low serum albumin, and any drug that displaces MTX from its protein [1]. NSAIDs influence the pharmacokinetics of MTX by lowering renal excretion of MTX, decreasing renal perfusion, and inhibiting renal transport proteins. The decrease in transport proteins leads to increased free MTX. Thus, concomitant administration of NSAIDs may be associated with an increased risk of toxicity [6]. In this case, our patient had been using NSAIDs for systemic pain.

There are a few case reports of MTX-induced cardiac conduction disturbances, but only one previously published case report exists of MTX-induced cardiomyopathy. This report describes a patient found to have a decrease in LV ejection fraction to 25% after mistaking her MTX dose of 5 mg/week to 5 mg/day [1]. However, this case did not describe fulminant cardiac failure as in our patient, as this patient actually presented due to pancytopenia. It is interesting that our patient had such dire cardiac effects without bone marrow suppression. Furthermore, it is unclear if our patient’s kidney and liver dysfunction were due to MTX toxicity or due to cardiac failure since both acutely improved following ECMO initiation.

However, two other cases of MTX toxicity describe mucosal lesions as the primary sign [2]. Upon further reading of these reports, both patients described had heart failure symptoms although these were presumed to be preexisting morbidities. One patient died of cardiopulmonary complications and the other had a reported LV ejection fraction of 10% upon admission. Certainly, these could have been cases of MTX-induced cardiomyopathy.

To support the belief that MTX caused cardiotoxicity in our patient, researchers have identified that cardiotoxicity can be induced with high-dose MTX in rats [7]. These researchers found that MTX-induced cardiac damage is evidenced by a distortion in the normal cardiac histological structure, with significant oxidative and nitrosative stress shown as a significant increase in NADPH oxidase-2, malondialdehyde, and nitric oxide levels, along with a decrease in reduced glutathione concentration and superoxide dismutase activity.

When considering other potential etiologies for our patient’s cardiomyopathy, it is worth noting that his echocardiography findings indicated this was an acute problem. Neither ventricle was dilated and both were normal thickness. His valves were normal. His pulmonary artery catheterization data indicated that he was not in a fluid overload state and did not have pulmonary hypertension. He did not have ischemic cardiomyopathy owing to negative coronary angiography.

Systemic sclerosis can have direct cardiac involvement manifesting as myocardial damage, fibrosis of the conduction system, pericardial, and less frequently, valvular disease [8]. When clinically manifested, cardiac involvement is thought to be an important prognostic factor for patients with systemic sclerosis, since profound microvascular disease is a pathognomonic feature of systemic sclerosis. Both vasospasm and structural alterations are present. Notably, previous research indicates that in patients who had cardiotoxicity owing to systemic sclerosis, there was only slightly impaired left ventricular function (mean LVEF 54.1 ± 9.0%) and cardiac biopsies demonstrated inflammation and fibrosis—neither of which concur with our patient’s data [9].

While we could not completely rule out a viral cardiomyopathy etiology despite a negative panel of 19 different viruses, the patient denied illness except his COVID diagnosis in which he had only mild symptoms. COVID has been shown to increase the risk of myocardial infarction [10] and cardiomyopathy [11]. A recent meta-analysis of 29 articles related to COVID and cardiomyopathy with a total of 1460 patients found severe LV dysfunction in 10% of patients. Hypertension, diabetes, obesity, hyperlipidemia, and ischemic heart disease were the most reported comorbidities among patients with COVID and cardiomyopathy, none of which our patient had. Finally, without any sign of inflammation on cardiac biopsy, viral myocarditis is virtually ruled out as this is currently the gold standard for diagnosis.

## Conclusion

After a process of elimination, the most likely cause of this patient’s acute decline and rapid recovery of bi-ventricular function was MTX toxicity. Leucovorin may have aided the reversal of MTX toxicity. It is unclear if the renal and hepatic impairments were owing to MTX or severe cardiomyopathy. However, both liver and renal function were normal immediately prior to his fulminant cardiac failure, decreasing the likelihood that these organs were adversely affected by direct MTX toxicity. It is also unknown why he did not have mucosal lesions or pancytopenia, as previously reported as early signs of MTX toxicity. What is known is that no etiology other than MTX cardiotoxicity was more likely, especially after reviewing the cardiac biopsy results. Finally, without VA-ECMO, this patient would have inevitably developed end-stage cardiac, renal, and hepatic dysfunction and died. Indeed, VA-ECMO was the ultimate survival modality, and we believe this case represents successful use of VA-ECMO for MTX cardiotoxicity.

## Data Availability

Not applicable.
